# Modified Ti-MWW Zeolite as a Highly Efficient Catalyst for the Cyclopentene Epoxidation Reaction

**DOI:** 10.3389/fchem.2020.585347

**Published:** 2020-10-09

**Authors:** Wen Tong, Jinpeng Yin, Luoyi Ding, Hao Xu, Peng Wu

**Affiliations:** Shanghai Key Laboratory of Green Chemistry and Chemical Processes, School of Chemistry and Molecular Engineering, East China Normal University, Shanghai, China

**Keywords:** Ti-zeolite, epoxidation reaction, cyclopentene, micro-environment, diffusion constrains

## Abstract

The liquid-phase epoxidation of cyclopentene (CPE) was performed in the Ti-zeolite/H_2_O_2_ catalytic system for the clean synthesis of cyclopentene oxide. Among all the Ti-zeolites (Ti-Beta, Ti-MOR, Ti-MCM-68, TS-1, TS-2, and Ti-MWW) investigated in the present study, Ti-MWW provided relatively lower CPE conversion of 13% due to the diffusion constrains but a higher CPO selectivity of 99.5%. The catalytic performance of Ti-MWW was significantly enhanced by piperidine (PI) treatment, with the CPE conversion and CPO selectivity increased to 97.8 and 99.9%, respectively. The structural rearrangement upon PI treatment converted the 3-dimensional (3D) MWW structure to a 2D lamellar one, which enlarged the interlayer space and greatly alleviated the diffusion constrains of cyclic cyclopentene. Furthermore, the newly constructed “open site” six-coordinated Ti active sites with PI as the ligand exhibited higher catalytic activity. The two factors contributed to more significant enhancement of the activity upon PI-assisted structural arrangement compared to the cases in linear alkenes.

## Introduction

Epoxy compounds, as important organic intermediates and chemical raw materials, can easily undergo ring-opening reactions with water, alcohols, amines, ammonia, or carboxylic acids to form a series of fine and bulk chemicals in the fields of petrochemical industry, polymer synthesis, and pharmaceuticals (Imamura et al., 1989; Yuan et al., 2011; Sharma et al., 2014; Jiang et al., 2019). The epoxidation of olefins is a typical way to produce epoxides. With the rapid development of the petrochemical industry, a large amount of cyclopentene (CPE) can be obtained from the C_5_ fraction of petroleum cracking, so the development of its downstream products has aroused increasing interests. In recent decades, many researchers focused on the catalytic oxidation reactions of cyclopentene, producing the corresponding alcohols, aldehydes, ketones, and acids (Xin et al., 2000; Jian et al., 2001; Dubkov et al., 2002; Guo et al., 2003; Yang et al., 2005; Yan and Xu, 2010). Moreover, several studies on cyclopentene epoxidation have also been reported (Hulea et al., 1998; Kluson et al., 2005). The main product of cyclopentene epoxidation is 1,2-epoxycyclopentane (CPO), which is widely used as the intermediate in medicine and organic synthesis (Milen et al., 2000; Huang et al., 2015; Seol et al., 2020).

Cyclopentene has been reported to be epoxidized by oxygen, hydrogen peroxide (H_2_O_2_), or organic peroxide, producing 1,2-epoxycyclopentane (Pramanik et al., 2007; Aleksandra, 2008; Maiti et al., 2008). However, the process using oxygen as the oxidant generally requires the addition of isobutyraldehyde and other co-oxidants to obtain a high yield (Qi et al., 2005; Mekrattanechai et al., 2018). In addition, the reaction system is too complex and difficult to control. The usage of organic peroxides could achieve high CPO selectivity, but the high price of organic peroxides would be a great issue. Hydrogen peroxide, with water as the sole by-product, serves as an environmentally friendly oxidant. However, H_2_O_2_ is a mild oxidant and thus needs appropriate catalysts for the CPE epoxidation reaction. Zhu et al. (2010) reported a three-phase transfer catalyst of phosphotungstic acid quaternary ammonium salt grafted by polystyrene-divinylbenzene to catalyze the CPE epoxidation with KCl as a co-catalyst, under solvent-free conditions, achieving a CPO yield of 96%. However, the cost of a phase transfer catalyst is relatively high. Yin et al. (2007) used nano-silver as the catalyst, giving an extremely low CPE conversion of 1.7–3.2%, which was not suitable for industrial production.

The titanosilicates are effective catalysts in liquid-phase epoxidation reactions, using H_2_O_2_ as the oxidant. Also, the Ti-zeolite/H_2_O_2_ catalytic system exhibited significant advantages, such as mild reaction conditions, high conversion, good selectivity, and environmental friendliness. The successful synthesis and application of TS-1 in the selective oxidation reactions are an important milestone in the field of zeolite catalysis after the industrial application of Y and ZSM-5 zeolite in the oil refining processes (Taramasso et al., 1983). As the first-generation Ti-zeolite, TS-1 has been extensively studied and widely used in various catalytic oxidation reactions, including olefin epoxidation, aromatic hydroxylation, aldehyde and ketone ammoxidation, and thiophene, alcohol, and amine oxidation, etc. (Spinace et al., 1995; Laha and Kumar, 2002; Kong et al., 2004). Afterward, numerous titanosilicates with different structural topologies have been reported such as microporous Ti-Beta (Wang et al., 2019), Ti-MWW (Wu et al., 2001), and mesoporous Ti-MCM-41 (Blasco et al., 1995). Very recently, extra-large pore Ti-zeolites with pores larger than the 12-member ring (MR), including Ti-ECNU-9 (Yang et al., 2018) and Ti-UTL (Liu et al., 2017; Shi et al., 2019), have been synthesized *via* post-modification strategy on layered zeolites and germanosilicates, respectively, which showed absolute advantages in processing bulky molecules.

The “five-membered ring” reaction mechanism is generally accepted in the Ti-zeolite/H_2_O_2_ catalytic system (Clerici and Ingallina, 1993). The tetrahedrally coordinated TiO_4_ species first interact with H_2_O_2_ to form the active intermediate species of Ti–O^α^ − *O*^β^ − H^end^, while the protic solvent (alcohol or water) in the reaction system coordinates with the titanium atom and forms a hydrogen bond with O^β^, thereby forming a five-membered ring intermediate. In addition, the hydrogen bonds can also be formed between Ti–O^α^ − *O*^β^ − H^end^ species and the neighboring Si–OH to stabilize Ti–O^α^ − *O*^β^ − H^end^ species (Ratnasamy and Srinivas, 2004). The O^α^ atom, directly connected to titanium atoms, has a stronger electrophilicity than O^β^. Thus, it can easily attack the double bond in the olefin molecules to transfer the active oxygen effectively and accomplish the epoxidation of olefins. Bearing the reaction mechanism in mind, the researchers have focused on modifying the microenvironment of Ti active sites with the purpose of improving the catalytic performance. The F atoms with strong electronegativity were introduced into the frameworks of Ti-MWW (Fang et al., 2017) and Ti-MOR zeolite (Yang et al., 2014), forming SiO_3/2_F groups, which imposed a strong electron pulling effect and improved the electropositivity of Ti active sites, thereby favoring the O^α^ transfer step and enhancing the catalytic performance. Also, numerous studies have shown that framework Ti species with a higher coordination number exhibited better catalytic performance in the epoxidation reactions. Xu et al. (2020) reported the one-step rapid synthesis of TS-1 with mononuclear TiO_6_ species affording a high turnover number value of 272 in the 1-hexene epoxidation reaction. Wu et al. (2016) illustrated that the mononuclear “TiO_6_” species showed the catalytic activity 2–3 times that of the “TiO_4_” species in TS-1 for the epoxidation of alkenes.

Among all the titanosilicates, Ti-MWW, with two sets of independent 10-MR pore channels, is fabricated from a layered precursor *via* interlayer condensation. One set is the sinusoidal 10-MR pore channel (0.4 × 0.51 nm) running within layers, and the other set is the 10-MR pore (0.40 × 0.55 nm) in the interlayer space (Wu et al., 2001). Although with relatively narrow pore opening, the unique lamellar structure of MWW-type titanosilicate provides the property of structural modification, such as delamination (Wu et al., 2004), interlayer expansion (Satoshi et al., 2011), and pillaring (Hao et al., 2016), to expose a larger surface area or enlarge the interlayer pore entrance. Moreover, the structural modifiable nature can also assist the modification of the microenvironment of Ti active sites by piperidine (PI) treatment. The transformation of a 3D structure to a 2D one occurred in the PI treatment and allowed the coordination of PI molecules to Ti atoms, forming the “open site” hexa-coordinated Ti active sites, which greatly improved the catalytic activity in the alkene epoxidation reactions (Xu et al., 2015).

Herein, with the purpose to find an appropriate catalyst for the CPE epoxidation reaction, we have investigated several Ti-zeolites, including Ti-Beta, Ti-MOR, Ti-MCM-68, TS-1, TS-2, and Ti-MWW, among which Ti-MWW showed relatively lower CPE conversion but higher CPO selectivity with acetonitrile (MeCN) as the solvent. Then, the microenvironment modification of Ti active sites, realized by PI-assisted structural rearrangement, dramatically enhanced the catalytic activity of Ti-MWW with H_2_O_2_ as the oxidant. Although comparable high catalytic activity has also been obtained by delaminated Ti-MWW (Wu et al., 2004), the condition for preparing the PI-modified Ti-MWW catalyst was milder and the introduction of PI molecules not only transformed the 3D structure to a more open 2D one but also constructed more active open-site Ti species. Additionally, the reaction parameters were also carefully investigated for the Ti-MWW catalyzed CPE epoxidation reaction, indicating it is a potential industrial catalyst for 1,2-epoxycyclopentane production.

## Materials and Methods

### Catalyst Preparation

#### Preparation of Ti-MWW

Ti-MWW was hydrothermally synthesized with boric acid as the crystallization agent and piperidine (PI) as the organic structural directing agent (OSDA). The synthetic gel with a molar composition of 1.0 SiO_2_: 0.05 TiO_2_: 0.67 B_2_O_3_: 1.4 PI: 19 H_2_O was hydrothermally crystallized at 443 K for 7 days under a rotation rate of 10 rpm. The obtained sample was filtered, washed with deionized water, and dried at 373 K overnight. The obtained two-dimensional Ti-MWW lamellar precursor was denoted as Ti-MWW-AM. Then, Ti-MWW-AM was refluxed in 2 M HNO_3_ aqueous solution at 413 K for 4 h with the liquid/solid mass ratio of 30 to remove partial OSDA molecules, most of the framework B atoms, and extra-framework Ti species. The acid-treated product was filtered, washed with deionized water, and dried at 373 K overnight, denoted as Ti-MWW-AT. Subsequently, Ti-MWW-AT was calcined at 823 K for 6 h to produce the 3D Ti-MWW.

#### Structural Rearrangement of Ti-MWW

The structural rearrangement of Ti-MWW was carried out in the PI aqueous solution with a composition of 1.0 SiO_2_: 1.0 PI: 10 H_2_O at 443 K for 24 h dynamically. The obtained 2D Ti-MWW containing PI in the framework was filtered, washed, and dried at 373 K overnight, denoted as R-Ti-MWW-PI. As a reference, R-Ti-MWW-PI was further calcined at 823 K for 6 h to remove PI molecules, denoted as R-Ti-MWW-PI-cal.

#### Synthesis of Different Titanosilicates

For control experiment, other titanosilicate catalysts, including Ti-Beta (Wang et al., 2019), Ti-MOR (Yang et al., 2014), Ti-MCM-68 (Kubota et al., 2008), TS-1 (Taramasso et al., 1983), and TS-2 (Reddy and Kumar, 1991) were prepared strictly according to the methods reported in the literatures.

### Characterization Methods

The X-ray diffraction (XRD) patterns were collected on a Rigaku Ultima IV diffractometer using Ni-filtered CuKα radiation (λ = 0.1541 nm). The voltage and current were 35 kV and 25 mA, respectively. The amount of Si and Ti was determined by inductively coupled plasma-atomic emission spectrometry (ICP-AES) on a Thermo IRIS Intrepid II XSP atomic emission spectrometer. The UV-Vis spectra were collected on a Shimadzu UV-2700 spectrophotometer by using BaSO_4_ as a reference. The FT-IR spectra were measured using the self-supported wafer by a Nicolet Nexus 670 FT-IR spectrometer with a resolution of 2 cm^−1^. The spectra in the framework vibration region (500–1,300 cm^−1^) were measured using KBr pellet technology. In order to eliminate the influence of absorbed water, all the samples were evacuated at 723 K for 3 h before measurement. Thermogravimetric (TG) analysis was carried out in a Netzsch Sta 4049 F3 apparatus in air with a heating rate of 10 K min^−1^ in the temperature range of 473–1,073 K. The solid-state ^29^Si MAS NMR spectra were recorded on a VARIAN VNMRS-400 MB NMR spectrometer using a 7.5-mm T3HX probe and single-pulse method at a frequency of 100.54 MHz and spinning rate of 3 KHz. [(CH_3_)_3_SiO]_8_SiO_12_ was used as the chemical shift reference. The surface and pore volume of the titanosilicates were determined by N_2_ physical sorption isotherms at 77 K using a BEL SORP instrument after degassing in vacuum at 473 K for 3 h. X-ray photoelectron spectroscopy (XPS) was measured using the Axis Ultra Imaging Photoelectron Spectrometer (Kratos Analytical Ltd.).

### Catalytic Experiments

The liquid-phase cyclopentene epoxidation reaction was performed in a glass reaction tube equipped with a condenser tube. In a typical run, 50 mg of catalyst, 10 mmol of cyclopentene, 10 mL of solvent, 10 mmol of H_2_O_2_ (30 wt.%), or *tert*-butyl hydroperoxide (70 wt.%, TBHP) was placed in the reaction tube and stirred at 333 K for 2 h. After reaction, the mixture was centrifuged to separate the spent catalyst. The generated products were identified by a GC-MS (Agilent 6890 Series GC System, 5937 network mass selective detector). The amount of the remaining substrates and the products was analyzed by a gas chromatograph (Shimadzu 2014, FID detector) equipped with an Rtx-Wax capillary column. The residual amount of H_2_O_2_ was determined by the titration method with 0.05 M Ce (SO_4_)_2_ aqueous solution.

## Results and Discussion

### Liquid-Phase Epoxidation of Cyclopentene Over Various Titanosilicates

#### Effect of the Topologies of the Titanosilicates

**Figure 1:**
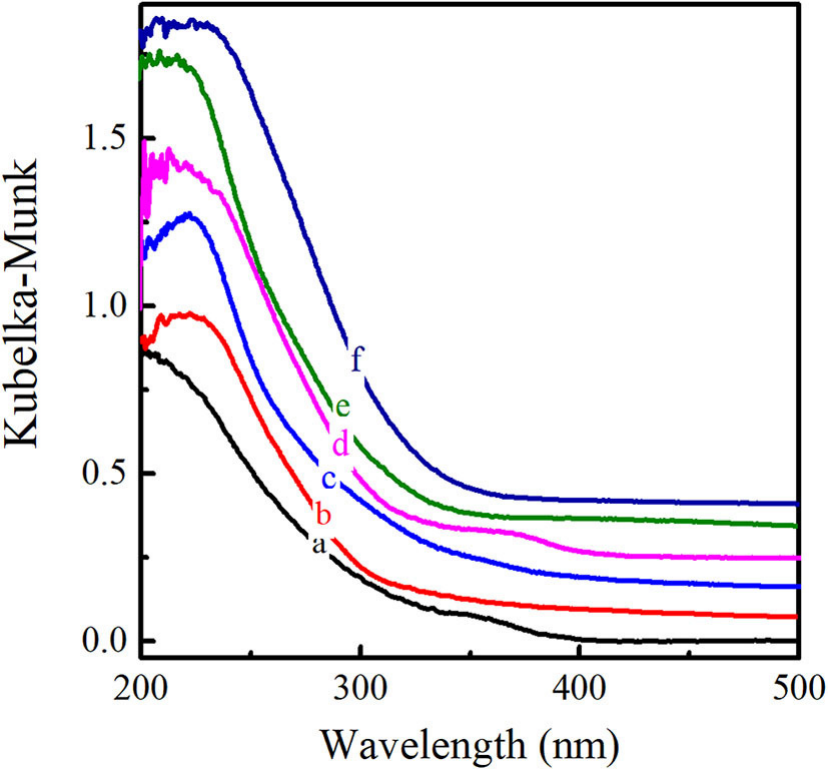
UV-visible diffuse reflectance spectra of Ti-MWW (a), Ti-Beta (b), Ti-MOR (c), Ti-MCM-68 (d), TS-1 (e), and TS-2 (f).

The XRD patterns of six titanosilicates with the topologies of MWW, ^*^BEA, MOR, MSE, MFI, and MEL, respectively, were displayed in Supplementary Figure 1, showing high crystallinity without any impurity. As shown in SEM images (Supplementary Figure 2), Ti-MWW possessed unique thin platelet morphology with the thickness of ~50 nm, which was quite different from Ti-Beta (50 nm), Ti-MOR (200–500 nm), Ti-MCM-68 (50–150 nm), TS-1 (200 nm), and TS-2 (50–100 nm). Moreover, the coordination states of Ti species were investigated by UV-Vis in Figure 1, indicating that Ti species were mainly tetrahedrally coordinated in the framework for all the titanosilicates. In order to select a suitable catalyst for the cyclopentene epoxidation process, the catalytic performance of the above titanosilicates was firstly investigated under the same reaction conditions (Table 1). The six titanosilicates showed different CPE conversions, with the order of Ti-MCM-68 > TS-1 > TS-2 > Ti-MWW > Ti-Beta > Ti-MOR. Ti-MCM-68 displayed the highest CPE conversion of 29.7%, while the least active Ti-MOR only converted 0.4% of CPE. TS-1 showed a slightly lower conversion than that of Ti-MCM-68, but the CPO selectivity was only 94.3% with considerable amount of 1,2-cyclopentanediol (CPD) as the by-product, indicating the easy occurrence of ring-opening reactions. Besides, Ti-Beta with 3D 12-MR pores showed a CPE conversion of 10.1% only higher than Ti-MOR. Although Ti-MWW exhibited moderate CPE conversion, the CPO selectivity was even higher than that of Ti-MCM-68. Additionally, cyclopent-2-enol (CPEL) and cyclopent-2-enone (CPEE) could also be examined in all the six titanosilicates. Considering that these titanosilicates contained various Ti contents, the specific activity in terms of TON value was also compared. Ti-MCM-68 showed the highest TON value of 311, followed by TS-1 and TS-2. Ti-MWW and Ti-Beta were inferior to them, and Ti-MOR showed the lowest TON value of 3. In fact, Ti-MWW has been reported previously to exhibit comparable activity to Ti-MCM-68, higher than TS-1, in the epoxidation of linear alkenes (Xu et al., 2020a). However, in the case of cyclic CPE molecules, they suffered severe diffusion constrains over the Ti-MWW catalyst, with relative narrower 10-MR pores than TS-1 and Ti-MCM-68, thus showing lower catalytic activity.

**Table 1:** A comparison of the catalytic performance of various titanosilicates in the cyclopentene epoxidation reaction with MeCN as the solvent^a^.

**Catalyst**	**Si/Ti^**b**^**	**TON**	**CPE^**c**^ conv. (%)**	**H**_****2****_**O**_****2****_ **(%)**		**Products distribution (%)**		
				**conv**.	**eff**.	**CPO^**c**^**	**CPD^**c**^**	**CPEL^**c**^ + CPEE^**c**^**
Ti-Beta	52	65	10.1	12.3	82.1	97.8	0.7	1.5
TS-1	33	118	28.6	32.9	87.0	94.3	5.6	0.1
TS-2	53	98	15.1	33.7	44.8	74.5	–	25.5
Ti-MWW	30	49	13.0	13.3	97.8	99.5	0.1	0.4
Ti-MOR	55	3	0.4	1.5	27.5	99.9	–	0.1
Ti-MCM-68	86	311	29.7	30.2	98.4	98.9	0.4	0.7

a *Reaction conditions: catalyst, 50 mg; cyclopentene, 10 mmol; H_2_O_2_ (30 wt.%), 10 mmol; MeCN, 10 mL; temp., 333 K; time, 2 h. TON in mol (mol Ti^−1^)*.

b *Molar ratio determined by ICP analysis*.

c *CPE, cyclopentene; CPO, cyclopentene oxide; CPD, 1, 2-cyclopentanediol; CPEL, cyclopent-2-enol; CPEE, cyclopent-2-enone*.

#### Influence of the Nature of Solvents

**Figure 2:**
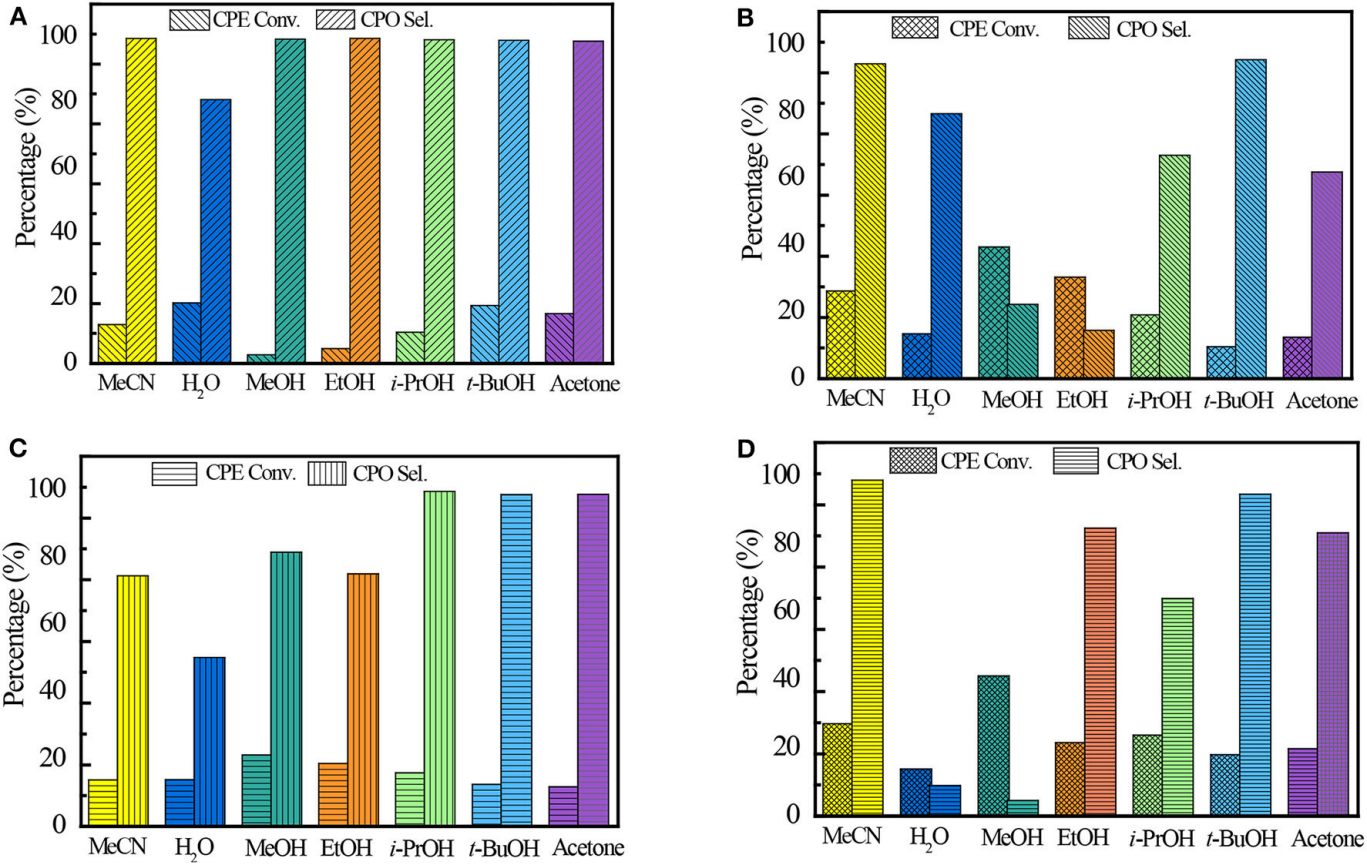
The catalytic performance of Ti-MWW **(A)**, TS-1 **(B)**, TS-2 **(C)**, and Ti-MCM-68 **(D)** in CPE epoxidation reaction with various solvents. Reaction conditions: catalyst, 50 mg; cyclopentene, 10 mmol, H_2_O_2_ (30 wt.%), 10 mmol; solvent, 10 mL; temp., 333 K; time, 2 h.

It is well-known that the solvent plays a very important role in the catalytic performance of epoxidation (Jan et al., 1997). The solvent effect on the Ti-zeolite/H_2_O_2_ system is comprehensive because it is related to various factors such as the protonicity of the solvent, the hydrophilicity/hydrophobicity of the titanosilicates, and the solubility of the substrates. Ti-MCM-68, TS-1, TS-2, and Ti-MWW, with relatively higher CPE conversion, were further investigated in various solvents, including MeCN, H_2_O, methanol (MeOH), ethanol (EtOH), isopropanol (*i*-PrOH), *tert*-butanol (*t*-BuOH), and acetone (Figure 2). In all the solvents, Ti-MWW exhibited extremely high CPO selectivity of >98.6% except H_2_O, indicating that CPO was very stable and prone to hydrolyze to CPD *via* the ring-opening side reaction only in the solvent of H_2_O (Figure 2A). The transformation of the 2D lamellar MWW precursor to the 3D structure *via* calcination would inevitably induce structural defects due to the imperfect interlayer condensation. The protic solvent molecules would easily adsorb in the pore channels of the 3D MWW framework and hindered the diffusion of substrate molecules. Thus, the aprotic solvent of MeCN, acetone, and the protic alcohol solvent with longer alkyl line (i.e., *t*-BuOH) showed higher activity. Among all the solvents, MeCN gave the highest selectivity of 99.5% compared to other alcohols (MeOH, EtOH, *i*-PrOH, and *t*-BuOH), which would promote the solvolysis of CPO to produce corresponding cyclopentanol ethers (2-methoxycyclopentanol, 2-ethoxycyclopentanol, 2-isopropoxycyclopentanol, and 2-(*tert*-butoxy)cyclopentanol) (Supplementary Table 1).

Unlike Ti-MWW, TS-1, and TS-2, with less defect sites, exhibited the highest CPE conversion of 43 and 23.2%, respectively, when using MeOH as the solvent. In the solvent of *t*-BuOH, TS-1 and TS-2 both exhibited high CPO selectivity of 95.6 and 98.6%, respectively. On the one hand, the relatively larger molecular size of *t*-BuOH in the pore channels may restrict the ring-opening side reactions because of a narrowed space. On the other hand, the much lower CPE conversion may also contribute to the higher selectivity. The undesired product of diols was also detected in aprotic solvents, which may be caused by the water formed in the epoxidation reaction (Supplementary Tables 2, 3).

In the case of Ti-MCM-68, the highest CPO selectivity of 98.9% was obtained in the solvent of MeCN due to the presence of defect sites in the structure. Although with similar solvent preference as Ti-MWW, the hydration of CPO to the by-product CPD in the solvent of H_2_O and MeOH over Ti-MCM-68 was more prominent than that over Ti-MWW, with the CPD selectivity of >90.9%, probably due to the residual Al-related active sites in the structure of Ti-MCM-68 catalyst (Supplementary Table 4).

#### A comparison of the Catalytic Performance Over a Series of Ti-MWW

Although Ti-MWW was not the most active one in the CPE epoxidation reaction among all the investigated Ti-zeolites, the selectivity was extremely high. Moreover, considering the possibility of modifying Ti-MWW with respect to both the structure and microenvironment of Ti active sites, Ti-MWW was further studied. The PI-assisted arrangement has been reported to be an effective way to enhance the catalytic performance of Ti-MWW zeolites in the epoxidation reactions of alkenes (Wu et al., 2016). Thus, in the present case of cyclopentene, PI-assisted structural modification was also performed. As shown in Table 2, with comparable Si/Ti ratio, the CPE conversion was remarkably enhanced from 13.0% for Ti-MWW to 97.8% for R-Ti-MWW-PI, with the CPO selectivity increased from 99.5 to 99.9% (Table 2, Nos. 1 and 2). In fact, the enhancement of the catalytic performance in CPE epoxidation was more significant compared to other alkene epoxidation reactions (Xu et al., 2015), probably because the cyclic CPE molecule encountered more severe diffusion constrains than other reported linear alkenes. The 3D Ti-MWW was transformed to 2D lamellar MWW structure upon the PI-assisted structural arrangement with the PI molecules intercalated into the interlayer space as well as the intralayer 10-MR pores, which enlarged the interlayer space compared to the original 10-MR pore channels and released the diffusion constrains, in spite the presence of PI molecules may also hinder the diffusion of substrates to some extent. Once the R-Ti-MWW-PI was calcined to remove the PI molecules and interlayer 10-MR pores were formed, the CPE conversion was decreased dramatically to 15.1%, which was slightly higher than that of the 3D Ti-MWW catalyst, while the CPO selectivity was decreased to 94.4% (Table 2, No. 3). As has been reported before in the case of linear alkenes (Xu et al., 2015), the calcined form of the PI-assisted MWW catalyst would show a much higher catalytic activity than the pristine 3D MWW structure because more “open-site” Ti species with H_2_O as the ligand formed in the structural reconstruction process and subsequent calcination. However, in the case of cyclic CPE molecules, the severe diffusion constraints weakened the benefit from the modified microenvironment of Ti active sites.

**Table 2:** The catalytic performance of Ti-MWW related catalysts in the cyclopentene epoxidation reaction^a^.

**No**.	**Catalyst**	**Si/Ti^**b**^**	**Si/B^**b**^**	**N content^**c**^ (wt.%)**	**PI/Ti**	**CPE^**d**^ conv. (%)**	**TON**	**H**_****2****_**O**_****2****_ **(%)**		**CPO^**d**^ sel. (%)**
								**conv**.	**eff**.	
1	Ti-MWW	30	69	–	–	13.0	49	13.3	97.8	99.5
2	R-Ti-MWW-PI	32	75	1.95	3.49	97.8	391	98.0	99.8	99.9
3	R-Ti-MWW-PI-cal	32	80	–	–	15.1	61	16.1	94.4	99.8
4	Ti-MWW-AM	22	14	2.51	3.17	0.3	1	21.2	1.5	99.2
5	Ti-MWW-AT	30	67	0.93	1.49	25.1	94	40.2	62.4	99.8

a *Reaction conditions: catalyst, 50 mg; cyclopentene, 10 mmol; H_2_O_2_ (30 wt.%), 10 mmol; MeCN, 10 mL; temp., 333 K; time, 2 h. TON in mol (mol Ti^−1^)*.

b *Molar ratio determined by ICP analysis*.

c *Determined by CHN analysis*.

d *CPE, cyclopentene; CPO, cyclopentene oxide*.

In the preparation of a highly active R-Ti-MWW-PI catalyst, the removal of PI molecules in the as-made Ti-MWW-AM structure *via* the successive acid treatment and calcination and then the reinsertion of PI molecules back into the MWW structure caused a waste of PI molecules and was uneconomic. Since the acid treatment in 2 M HNO_3_ aqueous solution could remove most of B atoms and the extra framework Ti species together with only part of the occluded OSDA molecules and the structure was still in 2D type (Wu et al., 2001), the acid-treated sample Ti-MWW-AT may show a similar catalytic behavior as that of R-Ti-MWW-PI. Before verifying this assumption, the structure, the amount of PI molecules, the porosity, and the state of Ti species in the framework were analyzed for all the Ti-MWW-related catalysts.

**Figure 3:**
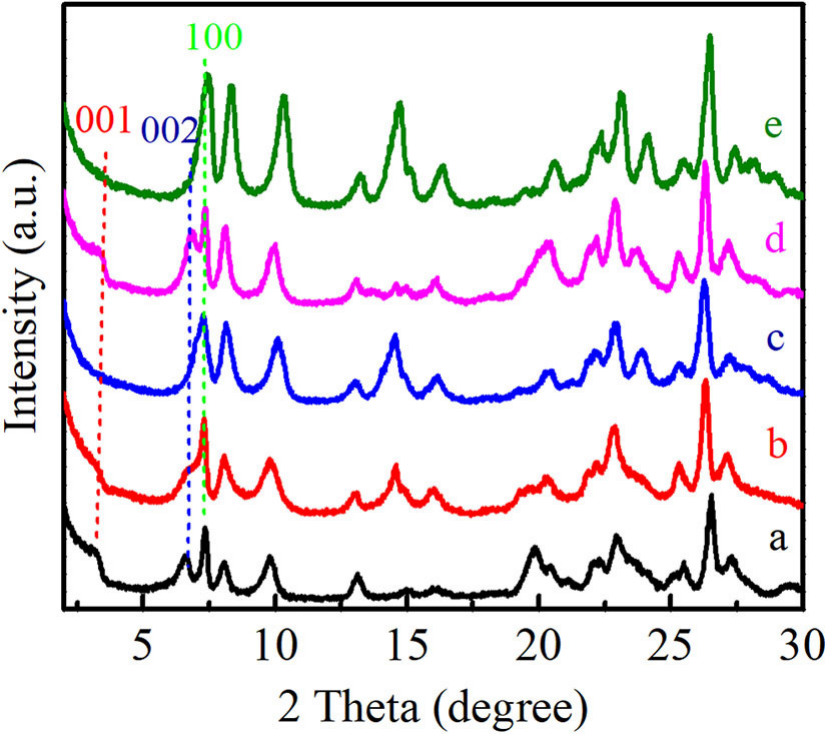
XRD patterns of Ti-MWW-AM (a), Ti-MWW-AT (b), Ti-MWW (c), R-Ti-MWW-PI (d), and R-Ti-MWW-PI-cal (e).

As shown in Figure 3, the XRD patterns showed that all of these Ti-MWW-related catalysts possessed the typical MWW topology. Ti-MWW-AM showed the characteristic layer-related 001 and 002 diffractions in the 2θ range of 3–7°, indicative of its 2D lamellar structure. After the acid treatment, the intensity of these two layer-related diffraction peaks decreased, because the partial OSDA removal disturbed the ordered layer-stacking in the pristine Ti-MWW-AM material. In spite of this, the Ti-MWW-AT still maintained the 2D structure. After further calcination, both of the 001 and 002 diffraction peaks disappeared, forming the 3D Ti-MWW structure. Upon the PI-assisted structural rearrangement, the layered-related 001 and 002 diffraction peaks were recovered for the R-Ti-MWW-PI material, due to structural transformation of the 3D to 2D MWW structure through the reinsertion of PI molecules into the framework. By removing the PI molecules *via* further calcination, the 2D structure was converted back into the 3D MWW structure, with the characteristic layer-related 001 and 002 peaks disappearing again.

**Figure 4:**
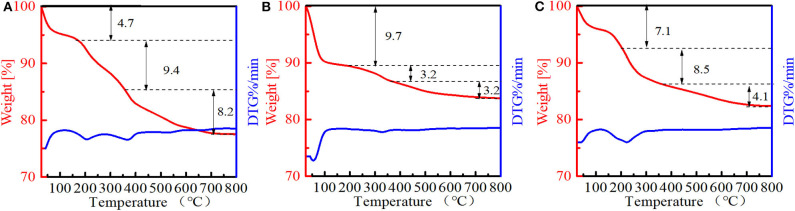
TGA analysis of Ti-MWW-AM **(A)**, Ti-MWW-AT **(B)**, and R-Ti-MWW-PI **(C)**.

The amounts of PI molecules occluded in the Ti-MWW-related structures were investigated by TG analysis, and the results are displayed in Figure 4. All the three PI-containing Ti-MWW catalysts, including Ti-MWW-AM, Ti-MWW-AT, and R-Ti-MWW-PI, showed three weight loss stages in the temperature range of 25–200°C, 200–350°C, and 350–800°C, attributed to the loss of physically absorbed water, the PI molecules occluded in the interlayer space, and the PI molecules located in the intralayer 10-MR pores, respectively. Meanwhile, a small part of weight loss at high temperatures could also include water from condensation of hydroxyl groups. The amount of the interlayer and intralayer PI molecules in Ti-MWW-AM material was 9.4 and 8.2%, respectively (Figure 4A). After the acid treatment, 65.9% of the interlayer PI molecules and 60.9% of the intralayer ones were extracted (Figure 4B). In the case of R-Ti-MWW-PI, the amounts of PI in the interlayer and intralayer space reached 8.5 and 4.1%, respectively (Figure 4C). According to Ti content and CHN elemental analysis, the molar ratios between the total PI and Ti amount of Ti-MWW-AM, Ti-MWW-AT, and R-Ti-MWW-PI were 3.17, 1.49, and 3.49 (Table 2), respectively.

The pore volume and surface area of all the Ti-MWW titanosilicates were determined by N_2_ sorption isotherms (Supplementary Figure 3), and the corresponding results are summarized in Table 3. The micropore volume and total surface area of Ti-MWW-AM was 0.01 cm^3^ g^−1^ and 65 m^2^ g^−1^, respectively. After the acid treatment, the micropore volume and total surface area increased to 0.05 cm^3^ g^−1^ and 184 m^2^ g^−1^ for Ti-MWW-AT. The occluded PI molecules were completely removed after further calcination, and the micropore volume and total surface area were significantly improved to 0.13 cm^3^ g^−1^ and 419 m^2^ g^−1^. After PI molecules were reinserted into the framework, the micropore volume and total surface area decreased to 0.04 cm^3^ g^−1^ and 140 m^2^ g^−1^ for R-Ti-MWW-PI. The removal of PI *via* calcination recovered the pore volume and surface area for 3D R-Ti-MWW-PI-cal. Since Ti-MWW-AT and R-Ti-MWW-PI possessed a comparable amount of intralayer PI molecules as revealed by the above TG analysis, the micropore volumes of the two 2D lamellar MWW structures, contributed by mainly intralayer 10-MR pores, were also very close. However, R-Ti-MWW-PI, containing more interlayer PI molecules, showed a lower total surface area than Ti-MWW-AT. Combining the results of TG and BET analysis, it is obvious that the PI-free 3D Ti-MWW samples possessed a higher pore-opening degree than those of PI-containing 2D structure Ti-MWW materials. For the three PI-containing materials, the pore-opening degree followed the order of Ti-MWW-AT > R-Ti-WWW-PI > Ti-MWW-AM.

**Table 3:** The physicochemical properties of Ti-MWW related catalysts.

**Sample**	**Pore volume (cm**^****3****^ **g**^****−1****^**)**			**SSA (m**^****2****^ **g**^****−1****^**)**		
	**${\text{V}}_{\text{total}}^{\text{a}}$**	**${\text{V}}_{\text{micro}}^{\text{b}}$**	**${\text{V}}_{\text{meso}}^{\text{c}}$**	**${\text{S}}_{\text{total}}^{\text{a}}$**	**S_**micro**_**	**${\text{S}}_{\text{ext}}^{\text{B}}$**
Ti-MWW-AM	0.33	0.01	0.32	65	4	61
Ti-MWW-AT	0.45	0.05	0.40	184	77	107
Ti-MWW	0.50	0.13	0.37	419	299	120
R-Ti-MWW-PI	0.42	0.04	0.38	140	75	65
R-Ti-MWW-PI-cal	0.49	0.14	0.35	445	335	110

a *Given by N_2_ adsorption at 77 K*.

b *Calculated by the BET method*.

c *Calculated by the t-plot method*.

**Figure 5:**
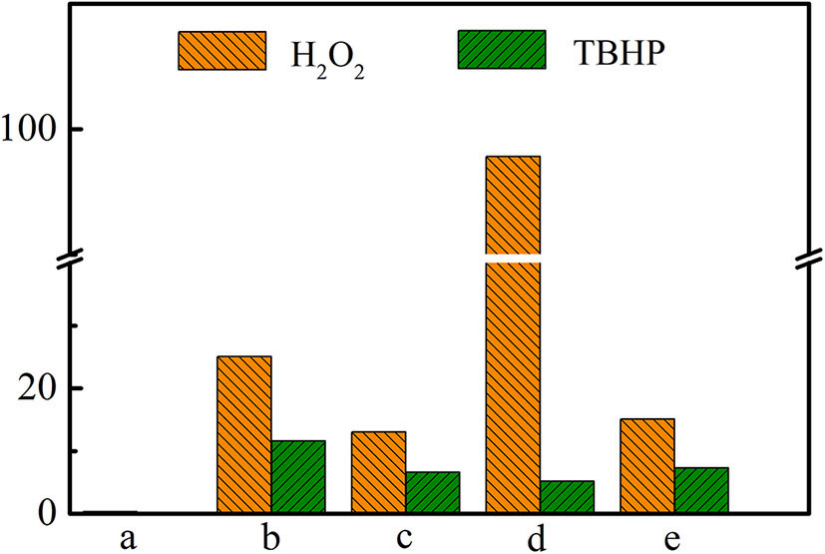
The catalytic performance of Ti-MWW-AM (a), Ti-MWW-AT(b), Ti-MWW (c), R-Ti-MWW-PI (d), and R-Ti-MWW-PI-cal (e) in the CPE epoxidation reaction with H_2_O_2_ or TBHP as an oxidant. Reaction conditions: catalyst, 50 mg; cyclopentene, 10 mmol, H_2_O_2_ (30 wt.%) or TBHP (70 wt.%), 10 mmol; MeCN, 10 mL; temp., 333 K; time, 2 h.

As has been shown in Table 2, Ti-MWW-AT exhibited the CPE conversion of 25.1% and CPO selectivity of 99.8%, showing a TON value much higher than that of Ti-MWW-AM, because the partial removal of PI molecules provided space for the diffusion of substrates. Also, the acid treatment could remove most of the boron atoms, and the final boron content was low with the Si/B ratio >67 (Table 2). As has been investigated in our recent study (Xu et al., 2020b), a further increase in the Si/B ratio to >600 for Ti-MWW, synthesized from the B containing system with a similar method in the present study, hardly alters the catalytic activity. Thus, the role of B atoms could be excluded. After further removal of the PI molecules *via* calcination, the catalytic performance of 3D Ti-MWW was inferior to that of Ti-MWW-AT, due to the formation of interlayer narrow 10-MR pore channels. However, the catalytic activity of Ti-MWW-AT was much lower than that of R-Ti-MWW-PI, although the pore-opening degree of Ti-MWW-AT was higher and they both contained PI molecules with a 2D lamellar structure. The PI molecules occluded inside the framework of Ti-MWW-AT and R-Ti-MWW-PI may impose different effects on the Ti active sites and result in distinct TON values. The bulky TBHP was also used as the oxidant to investigate the catalytic activity of these Ti-MWW-related catalysts (Figure 5). All of the catalysts suffered a decrease in catalytic activity due to the severe diffusion constrains for the bulky TBHP molecules. Among them, Ti-MWW-AT exhibited the highest CPE conversion of 11.7%, even higher than that of R-Ti-MWW-PI (5.3%), which was contrary to the case using H_2_O_2_ as the solvent. The catalytic advantage of R-Ti-MWW-PI benefiting from the hexa-coordinated Ti active sites was dramatically weakened when using the bulky TBHP as the oxidant because pore-blocking effect of PI molecules was dominated in this case.

#### Characterization of the Microenvironment of Ti Active Sites

**Figure 6:**
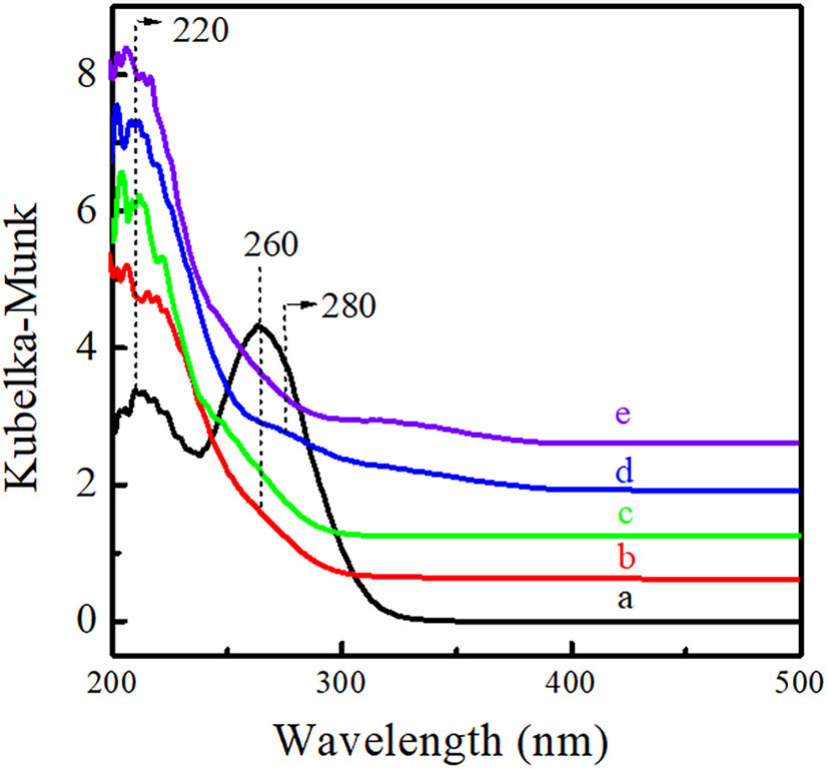
UV-visible diffuse reflectance spectra of Ti-MWW-AM (a), Ti-MWW-AT (b), Ti-MWW (c), R-Ti-MWW-PI (d), and R-Ti-MWW-PI-cal (e).

The microenvironment of Ti active sites was investigated *via* UV-Vis, FT-IR, and XPS spectra. As shown in Figure 6, Ti-MWW-AM displayed a main absorption band at 260 nm attributed to the extra-framework six-coordinated Ti species, together with a shoulder band at 220 nm, attributed to the framework TiO_4_ species. Both Ti-MWW-AT and Ti-MWW showed an absorption band at 220 nm attributed to TiO_4_ species, indicating that the extra-framework TiO_6_ species could be completely removed by the acid treatment. Besides the main band at 220 nm, R-Ti-MWW-PI also displayed a relatively weak shoulder band at 280 nm attributed to PI-coordinated Ti species, which was different from Ti-MWW-AT. After calcination, the weak absorption peak at 280 nm nearly disappeared for R-Ti-MWW-PI-cal. Moreover, the minor bands around 320–330 nm in the spectra of R-Ti-MWW-PI and R-Ti-MWW-PI-cal were attributed to TiO_2_ anatase formed through the aggregation of neighboring surface Ti species (Jarian et al., 2011). Therefore, the highest activity in the CPE epoxidation reaction when R-Ti-MWW-PI was used as the catalyst may be closely related to the coordination state of the Ti active sites.

The characteristic 960-cm^−1^ band in the IR spectra is generally accepted as the proof for the framework TO_4_ species, although the accurate attribution is still a debate. However, the presence of physically adsorbed H_2_O in the zeolite framework with silanol groups also contributes to the 960-cm^−1^ band. Thus, the samples were evacuated at elevated temperature before the FT-IR measurement to remove H_2_O, which would also remove the occluded PI molecules. Only the FT-IR spectra of Ti-MWW and R-Ti-MWW-PI-cal without PI molecules were measured (Supplementary Figure 4), and both of them showed the characteristic band at 960 cm^−1^, indicating the presence of framework TiO_4_ species.

**Figure 7:**
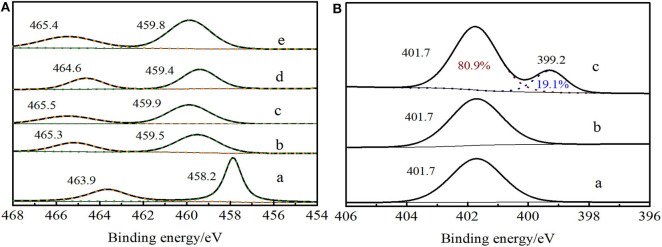
Ti 2p XPS spectra **(A)** of Ti-MWW-AM (a), Ti-MWW-AT (b), Ti-MWW (c), R-Ti-MWW-PI (d), and R-Ti-MWW-PI-cal (e) and N 1s XPS spectra **(B)** of Ti-MWW-AM (a), Ti-MWW-AT (b), and R-Ti-MWW-PI (c).

In Ti 2p XPS spectra of Ti-MWW (Figure 7Ac), it exhibited the signals at 459.9 eV and 465.5 eV, which were attributed to Ti 2p_3/2_ and Ti 2p_1/2_, respectively. For Ti-MWW-AM (Figure 7Aa), the presence of extra-framework Ti species resulted in a lower binding energy of 458.2 and 463.9 eV. Moreover, the binding energy shifted to 459.5 and 465.3 eV for Ti-MWW-AT after the removal of extra-framework Ti species *via* acid treatment (Figure 7Ab). The PI treatment reduced the binding energy from 459.9 eV for Ti-MWW to 459.4 eV for R-Ti-MWW-PI (Figure 7Ad), indicating that the charge distribution of the Ti ions in R-Ti-MWW-PI became more negative. According to the previous report (Xu et al., 2015), the broad peak around 459.4 eV was associated with a new six-coordinated Ti species with the PI molecule as a ligand and another six-coordinated Ti species with two water ligands. Then, the removal of organic PI ligands leads to the increase in binding energy values to 459.8 eV, because the PI ligands were replaced by water molecules and some framework TiO_4_ species were also restored upon calcination (Figure 7Ae). Although both with PI molecules in the framework, Ti-MWW-AM and R-Ti-MWW-PI showed different Ti 2p XPS spectra, meaning different microenvironments of Ti active sites. The difference indicated that PI molecules in the Ti-MWW-AM structure did not coordinate with Ti active sites and only filled the pore channels. This was further improved by the N 1s XPS spectra. In the N 1s XPS spectra, Ti-MWW-AM, Ti-MWW-AT, and R-Ti-MWW-PI all showed a binding energy value of 401.7 eV. However, only R-Ti-MWW-PI showed a peak with binding energy of 399.2 eV with the area ratio of 19.1%, indicating that PI may coordinate with the Ti active sites by N atoms (Figure 7B). Therefore, the coordinated PI/Ti ratio was 0.66 according to the total PI and Ti amount calculated by CHN and ICP analysis.

The interaction between PI molecules and Ti active sites was distinct for Ti-MWW-AT and R-Ti-MWW-PI as revealed by the band at 280 nm in the UV-vis spectra and different binding energy values in the XPS spectra. The PI molecules in the synthetic gel hardly coordinated with Ti active sites and constructed mainly “close-site” Ti active sites. In contrast, the structural rearrangement with alkaline PI solution at elevated temperature could cleave the Si–O–Ti bonds and create numerous “open-site” Ti active sites and the PI molecules could also serve as a ligand for the Ti atoms by N atoms. In a recent study reported by our group (Yin et al., 2020), the high catalytic activity of PI-modified Ti-MWW was mainly because the PI-coordinated TiO_6_ species could accelerate the activation of H_2_O_2_ rather than favoring the transformation of active O atoms in Ti–O–O–H intermediate to the alkene molecules.

### Effect of Reaction Parameters on Cyclopentene Epoxidation Over Ti-MWW and R-Ti-MWW-PI

#### Effect of the Catalyst Amount on Cyclopentene Epoxidation

**Figure 8:**
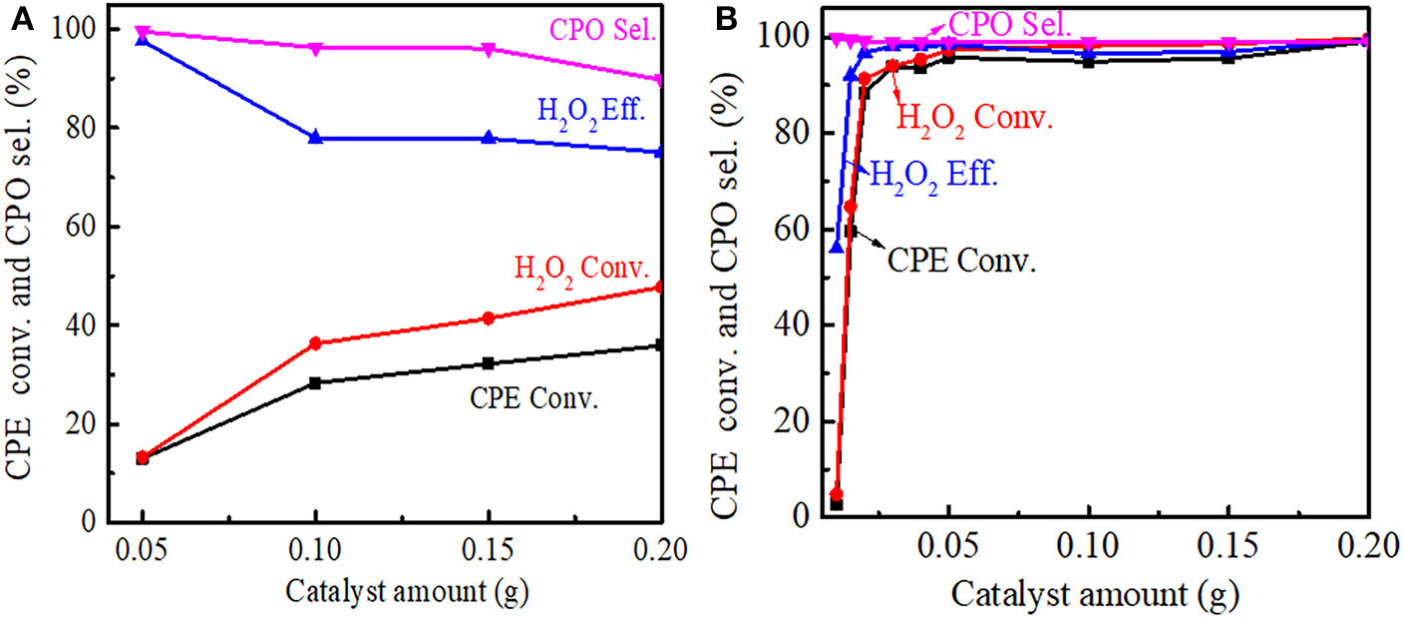
Dependence of CPE conversion, H_2_O_2_ conversion, H_2_O_2_ efficiency, and CPO selectivity on the amount of Ti-MWW **(A)**, and R-Ti-MWW-PI **(B)**. Reaction conditions: CPE, 10 mmol; H_2_O_2_ (30 wt.%), 10 mmol; MeCN, 10 mL; temp., 333 K; time, 2 h.

The effect of the catalyst amount on the cyclopentene epoxidation over Ti-MWW and R-Ti-MWW-PI was investigated, and the results are shown in Figure 8. With the Ti-MWW amount increasing from 50 to 150 mg, the CPE and H_2_O_2_ conversion also increased from 13.0 to 32.2% and from 13.3 to 41.4%, respectively (Figure 8A). Further increasing the Ti-MWW amount to 200 mg, both the CPE and H_2_O_2_ conversion increased slowly. However, with the increase in Ti-MWW amount, both the CPO selectivity and H_2_O_2_ efficiency gradually decreased. Although increasing the Ti-MWW amount favored the reactant conversion, the hydrolysis of CPO was also promoted due to the introduction of more Ti-related Lewis acid sites (Bittar et al., 1992). Comparatively, for R-Ti-MWW-PI, when the catalyst amount increased to 30 mg, both the CPE and H_2_O_2_ conversion reached nearly 90%, and they maintained at a very high level from 50 to 200 mg, almost close to 100% (Figure 8B). Under the same reaction conditions, the catalytic activity over R-Ti-MWW-PI was greatly enhanced compared to Ti-MWW. Thus, 50 mg of R-Ti-MWW-PI was enough to achieve high catalytic activity.

#### Effect of the Reaction Time on Cyclopentene Epoxidation

**Figure 9:**
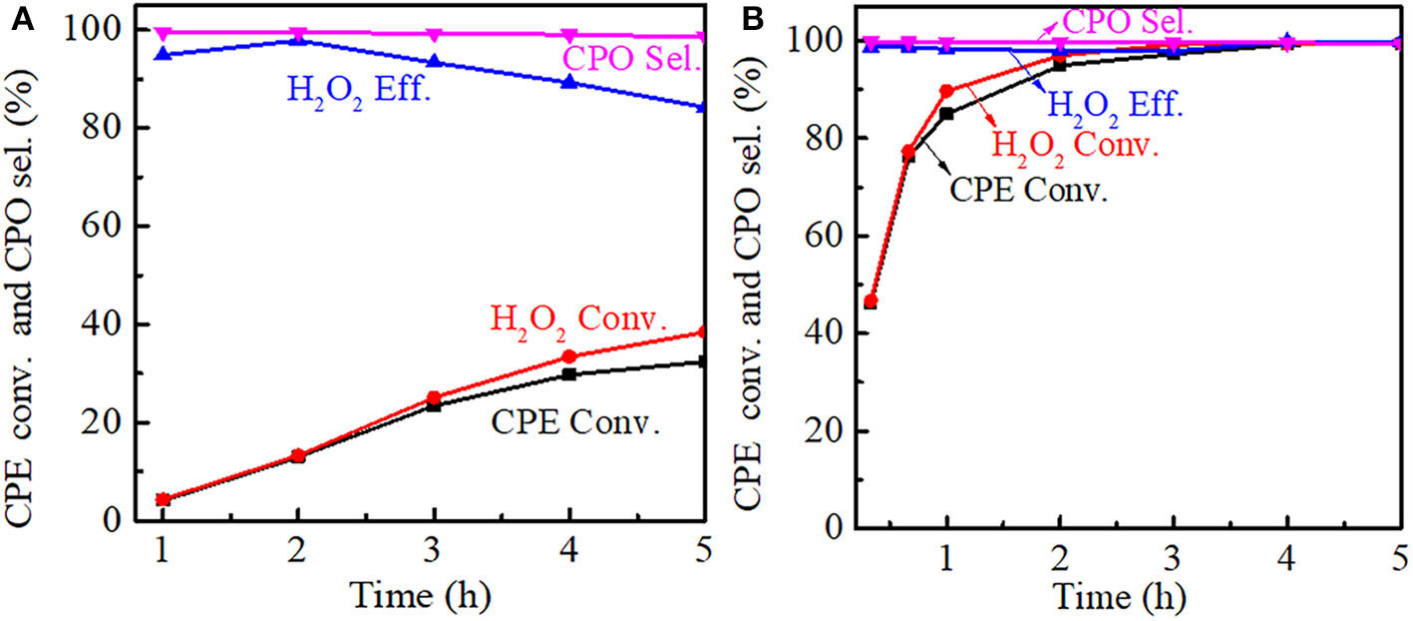
Dependence of CPE conversion, H_2_O_2_ conversion, H_2_O_2_ efficiency, and CPO selectivity on the reaction time over Ti-MWW **(A)** and R-Ti-MWW-PI **(B)**. Reaction conditions: catalyst, 50 mg; CPE, 10 mmol; H_2_O_2_ (30 wt.%), 10 mmol; MeCN, 10 mL; temp., 333 K.

The effect of reaction time on the cyclopentene epoxidation was investigated at 333 K over Ti-MWW and R-TI-MWW-PI, respectively. As shown in Figure 9A, prolonging reaction time from 0.5 to 5 h, both the CPE and H_2_O_2_ conversion increased for Ti-MWW, whereas the CPO selectivity was maintained stable at about 99%. The H_2_O_2_ efficiency reached a maximum value of 99.3% at 2 h and gradually decreased as the reaction time was prolonged, indicating the occurrence of the non-productive decomposition of H_2_O_2_. In the case of R-Ti-MWW-PI, the CPE conversion at a reaction time of 0.5 h exceeded that over Ti-MWW at a reaction time of 5 h (Figure 9B). For R-Ti-MWW-PI, both the CPE and H_2_O_2_ conversion increased with the reaction time prolonged, and the CPO selectivity as well as the H_2_O_2_ efficiency could be maintained at a high level.

#### Effect of the Reaction Temperature on Cyclopentene Epoxidation

**Figure 10:**
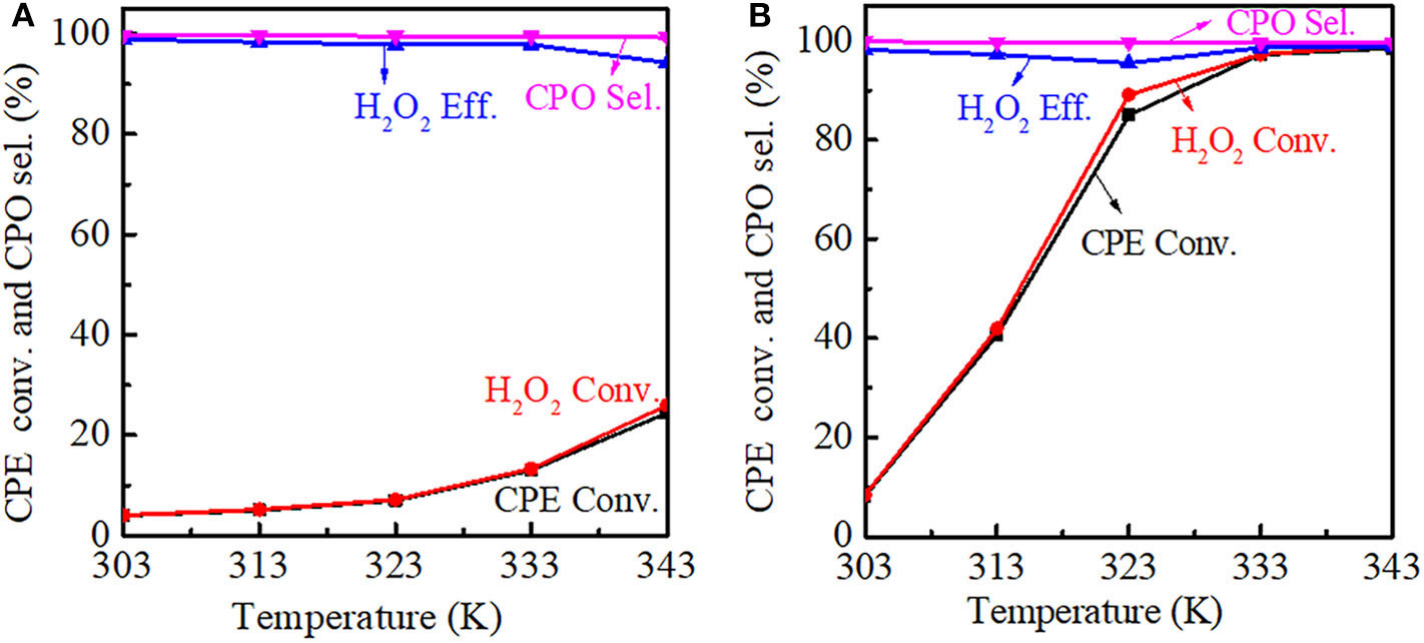
Dependence of CPE conversion, H_2_O_2_ conversion, H_2_O_2_ efficiency, and CPO selectivity at different reaction temperatures over Ti-MWW **(A)** and R-Ti-MWW-PI **(B)**. Reaction conditions: catalyst, 50 mg; CPE, 10 mmol; H_2_O_2_ (30 wt.%), 10 mmol; MeCN, 10 mL; time, 2 h.

The reaction temperature had a significant influence on the catalytic performance. For Ti-MWW, both the CPE and H_2_O_2_ conversion increased with the reaction temperature increasing, meanwhile the CPO selectivity was basically unchanged. However, the H_2_O_2_ efficiency decreased gradually from 313 to 343 K, indicating that H_2_O_2_ ineffective decomposition was accelerated at a higher reaction temperature. Also, it can be seen that as the reaction temperature increased, the growth rate of the conversion was improved and the CPE conversion was increased to 24.4% at 343 K (Figure 10A). For R-Ti-MWW-PI, the CPE and H_2_O_2_ conversion was greatly increased to nearly 100% at 343 K, and the CPO selectivity was maintained at 99.9% (Figure 10B).

The regeneration and recycling of R-Ti-MWW-PI are shown in Supplementary Figure 5. The CPE conversion decreased gradually by 3–8% after each reaction run while the CPO selectivity was maintained at 99.9%. After six runs, it was regenerated by calcination and PI treatment again, and the activity was restored in the seventh run.

## Conclusions

For the epoxidation of cyclopentene, Ti-MWW provided relatively lower CPE conversion due to the diffusion constrains. However, it showed the advantages in product selectivity in the aprotic solvent like MeCN. The structural rearrangement upon PI treatment converted the 3D MWW structure to a 2D lamellar one, which enlarged the interlayer space and greatly alleviated the diffusion constrains of cyclic cyclopentene. Besides, the newly formed hexa-coordinated Ti active species, bearing PI molecules as the ligand, exhibited higher catalytic activity. Moreover, the enhancement of the catalytic performance in cyclic CPE epoxidation by 7.5 times through the PI-assisted structural rearrangement was the most significant compared to other linear alkenes.

## Data Availability Statement

All datasets presented in this study are included in the article/Supplementary Material.

## Author Contributions

PW conceived and planned the research. WT prepared the catalysts and performed the characterization and catalytic experiments. JY and LD contributed to the catalyst preparation. WT and HX prepared the manuscript with contributions from the other authors.

## Conflict of Interest

The authors declare that the research was conducted in the absence of any commercial or financial relationships that could be construed as a potential conflict of interest.
