# Supplementary material for: Modified Ti-MWW Zeolite as a Highly Efficient Catalyst for the Cyclopentene Epoxidation Reaction
Source: Front Chem. 2020 Oct 9;8:585347. doi: 10.3389/fchem.2020.585347 (PMC7581914; doi:10.3389/fchem.2020.585347)
Supplement: Supplementary file 1 [file Data_Sheet_1.DOCX]

Supplementary Material

# Supplementary Data

**Supplementary Figure 1.** XRD patterns of Ti-MWW (a), Ti-Beta (b), Ti-MOR (c), Ti-MCM-68 (d), TS-1 (e) and TS-2 (f).

**Supplementary Figure 2.** SEM images of Ti-MWW (a), Ti-Beta (b), Ti-MOR (c), Ti-MCM-68 (d), TS-1 (e) and TS-2 (f).

**Supplementary Figure 3.** N_2_ adsorption-desorption isotherms of Ti-MWW-AM (a), Ti-MWW-AT (b), Ti-MWW (c), R-Ti-MWW-PI (d) and R-Ti-MWW-PI-cal (e).

**Supplementary Figure 4**. FT-IR spectra in framework vibration region of Ti-MWW (a) and R-Ti-MWW-PI-cal (b).

**Supplementary Figure 5**. The recycling and regeneration of R-Ti-MWW-PI in cyclopentene epoxidation.

**Supplementary Table 1.** A comparison of the catalytic performance over Ti-MWW in the cyclopentene epoxidation in different solvents.^a^

**Supplementary Table 2.** A comparison of the catalytic performance over TS-1 in the cyclopentene epoxidation in different solvents.^a^

**Supplementary Table 3.** A comparison of the catalytic performance over TS-2 in the cyclopentene epoxidation in different solvents.^a^

**Supplementary Table 4.** A comparison of the catalytic performance over Ti-MCM-68 in the cyclopentene epoxidation in different solvents.^a^

**Supplementary Table 5.** The results of CHN analysis.

# Supplementary Figures and Tables

## Supplementary Figures





**Supplementary Figure 1.** XRD patterns of Ti-MWW (a), Ti-Beta (b), Ti-MOR (c), Ti-MCM-68 (d), TS-1 (e) and TS-2 (f).


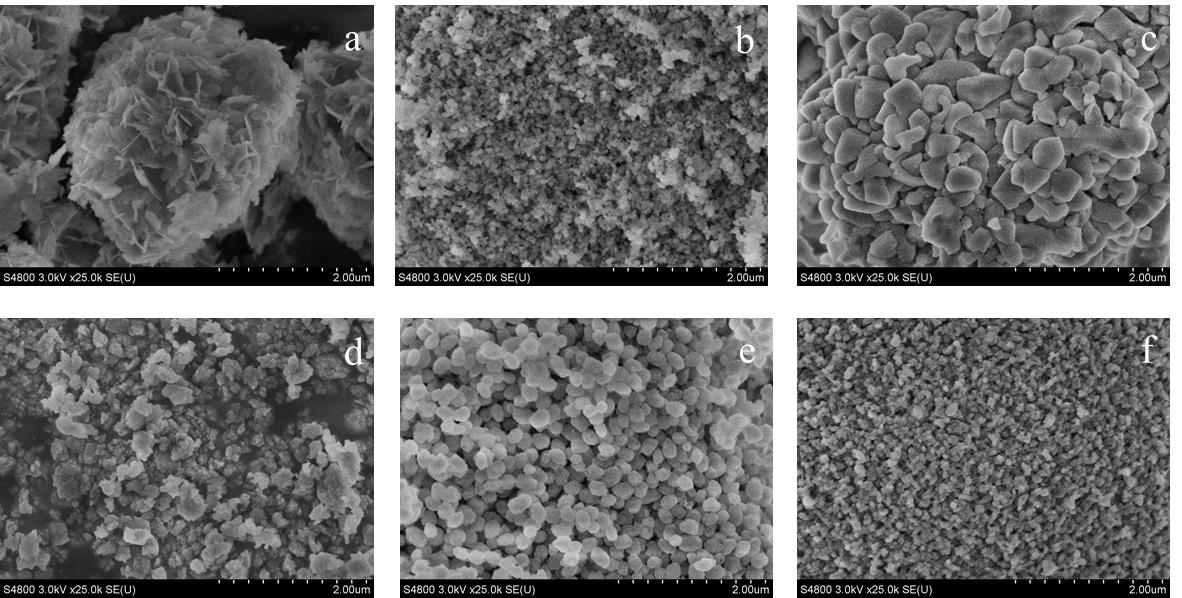


**Supplementary Figure 2.** SEM images of Ti-MWW (a), Ti-Beta (b), Ti-MOR (c), Ti-MCM-68 (d), TS-1 (e) and TS-2 (f).


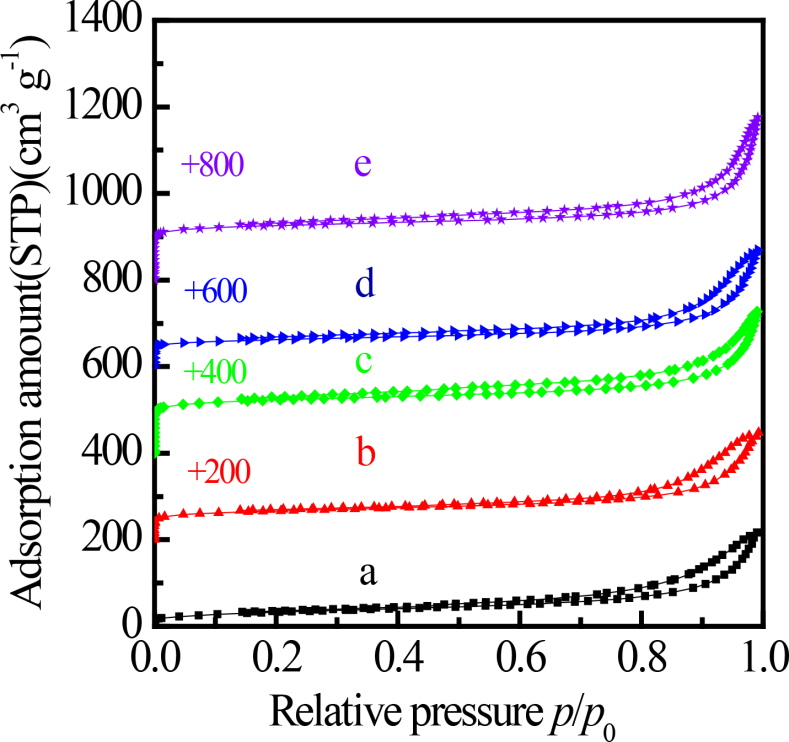


**Supplementary Figure 3.** N_2_ adsorption-desorption isotherms of Ti-MWW-AM (a), Ti-MWW-AT (b), Ti-MWW (c), R-Ti-MWW-PI (d) and R-Ti-MWW-PI-cal (e).





**Supplementary Figure 4**. FT-IR spectra in framework vibration region of Ti-MWW (a) and R-Ti-MWW-PI-cal (b).





**Supplementary Figure 5**. The recycling and regeneration of R-Ti-MWW-PI in cyclopentene epoxidation. The catalyst was washed by MeCN after each reaction run and finally regenerated by calcination and PI treatment again. The CPO selectivity was maintained at 99.9%.

## Supplementary Tables

**Supplementary Table 1.** A comparison of the catalytic performance over Ti-MWW in the cyclopentene epoxidation in different solvents.^a^

| Catalyst | Solvent | CPE ^b^  Conv. (%) | H_2_O_2_ (%) | |  | Products distribution ^c^ (%) | | | |
| --- | --- | --- | --- | --- | --- | --- | --- | --- | --- |
|  |  |  | Conv. | Eff. |  | CPO | CPD | others |  |
| Ti-MWW | MeCN | 13.0 | 13.3 | 97.8 |  | 99.5 | 0.1 | 0.4 |  |
|  | H_2_O | 20.2 | 28.3 | 71.4 |  | 80.8 | 19.1 | 0.1 |  |
|  | MeOH | 2.9 | 15.7 | 18.7 |  | 99.3 | - | 0.7 |  |
|  | EtOH | 4.9 | 13.3 | 36.7 |  | 99.5 | - | 0.5 |  |
|  | *i*-PrOH | 10.4 | 30.8 | 33.9 |  | 99.1 | 0.1 | 0.8 |  |
|  | *t*-BuOH | 19.3 | 38.9 | 49.5 |  | 98.9 | 0.1 | 1.0 |  |
|  | Acetone | 16.6 | 28.1 | 59.1 |  | 98.6 | 1.1 | 0.3 |  |

^a^ Reaction conditions: catalyst, 50 mg; cyclopentene, 10 mmol, H_2_O_2_ (30 wt.%), 10 mmol; solvent, 10 mL; temp., 333 K; time, 2 h. ^b^ CPE, Cyclopentene. ^c^ CPO, cyclopentene oxide; CPD, 1,2-cyclopentanediol; others, cyclopent-2-enol, cyclopent-2-enone and 2-methoxycyclopentanol for MeOH, 2-ethoxycyclopentanol for EtOH, 2-isopropoxycyclopentanol for *i*-PrOH and 2-(*tert*-butoxy)cyclopentanol for *t*-BuOH.

**Supplementary Table 2.** A comparison of the catalytic performance over TS-1 in the cyclopentene epoxidation in different solvents.^a^

| Catalyst | Solvent | CPE ^b^  Conv. (%) | H_2_O_2_ (%) | |  | Products distribution ^c^ (%) | | | |
| --- | --- | --- | --- | --- | --- | --- | --- | --- | --- |
|  |  |  | Conv. | Eff. |  | CPO | CPD | others |  |
| TS-1 | MeCN | 28.6 | 32.9 | 87.0 |  | 94.3 | 5.6 | 0.1 |  |
|  | H_2_O | 14.6 | 20.9 | 70.1 |  | 79.4 | 20.4 | 0.2 |  |
|  | MeOH | 43.0 | 57.7 | 74.5 |  | 22.2 | 5.2 | 72.6 |  |
|  | EtOH | 33.2 | 40.3 | 82.3 |  | 14.4 | 7.2 | 78.4 |  |
|  | *i*-PrOH | 20.8 | 29.3 | 71.1 |  | 66.9 | 4.3 | 28.8 |  |
|  | *t*-BuOH | 10.4 | 23.8 | 43.5 |  | 95.6 | 0.1 | 4.3 |  |
|  | Acetone | 13.5 | 28.9 | 46.7 |  | 61.9 | 37.2 | 0.9 |  |

^a^ Reaction conditions: catalyst, 50 mg; cyclopentene, 10 mmol, H_2_O_2_ (30 wt.%), 10 mmol; solvent, 10 mL; temp., 333 K; time, 2 h. ^b^ CPE, Cyclopentene. ^c^ CPO, cyclopentene oxide; CPD, 1,2-cyclopentanediol; others, cyclopent-2-enol, cyclopent-2-enone and 2-methoxycyclopentanol for MeOH, 2-ethoxycyclopentanol for EtOH, 2-isopropoxycyclopentanol for *i*-PrOH and 2-(*tert*-butoxy)cyclopentanol for *t*-BuOH.

**Supplementary Table 3.** A comparison of the catalytic performance over TS-2 in the cyclopentene epoxidation in different solvents.^a^

| Catalyst | Solvent | CPE ^b^  conv. (%) | H_2_O_2_ (%) | |  | Products distribution ^c^ (%) | | |
| --- | --- | --- | --- | --- | --- | --- | --- | --- |
|  |  |  | conv. | eff. |  | CPO | CPD | others |
| TS-2 | MeCN | 15.1 | 33.7 | 44.8 |  | 74.5 | - | 25.5 |
|  | H_2_O | 15.2 | 19.4 | 78.3 |  | 50.2 | 48.9 | 0.9 |
|  | MeOH | 23.2 | 32.9 | 70.4 |  | 81.5 | 1.6 | 16.9 |
|  | EtOH | 20.4 | 34.9 | 58.5 |  | 75.1 | - | 24.9 |
|  | *i*-PrOH | 17.4 | 22.9 | 75.6 |  | 99.6 | - | 0.4 |
|  | *t*-BuOH | 13.7 | 27.3 | 50.2 |  | 98.6 | 0.3 | 1.1 |
|  | Acetone | 12.9 | 22.6 | 57.2 |  | 98.7 | 1.0 | 0.3 |

^a^ Reaction conditions: catalyst, 50 mg; cyclopentene, 10 mmol, H_2_O_2_ (30 wt.%), 10 mmol; solvent, 10 mL; temp., 333 K; time, 2 h. ^b^ CPE, cyclopentene. ^c^ CPO, cyclopentene oxide; CPD, 1,2-cyclopentanediol; others, cyclopent-2-enol, cyclopent-2-enone and 2-methoxycyclopentanol for MeOH, 2-ethoxycyclopentanol for EtOH, 2-isopropoxycyclopentanol for *i*-PrOH and 2-(*tert*-butoxy)cyclopentanol for *t*-BuOH.

**Supplementary Table 4.** A comparison of the catalytic performance over Ti-MCM-68 in the cyclopentene epoxidation in different solvents.^a^

| Catalyst | Solvent | CPE ^b^  conv. (%) | H_2_O_2_ (%) | |  | Products distribution ^c^ (%) | | |  |
| --- | --- | --- | --- | --- | --- | --- | --- | --- | --- |
|  |  |  | conv. | eff. |  | CPO | CPD | others | |
| Ti-MCM-68 | MeCN | 29.7 | 30.2 | 98.4 |  | 98.9 | 0.4 | 0.7 | |
|  | H_2_O | 15.1 | 15.5 | 97.7 |  | 9.0 | 90.9 | 0.1 | |
|  | MeOH | 45.0 | 57.1 | 78.7 |  | 4.6 | - | 95.4 | |
|  | EtOH | 23.6 | 38.5 | 61.2 |  | 84.8 | 1.9 | 13.3 | |
|  | *i*-PrOH | 26.0 | 50.4 | 51.6 |  | 64.1 | 12.3 | 23.6 | |
|  | *t*-BuOH | 19.7 | 40.4 | 48.7 |  | 94.8 | 3.1 | 2.1 | |
|  | Acetone | 21.6 | 36.9 | 58.5 |  | 83.4 | 16.3 | 0.3 | |

^a^ Reaction conditions: catalyst, 50 mg; cyclopentene, 10 mmol, H_2_O_2_ (30 wt.%), 10 mmol; solvent, 10 mL; temp., 333 K; time, 2 h. ^b^ CPE, cyclopentene. ^c^ CPO, cyclopentene oxide; CPD, 1,2-cyclopentanediol; others, cyclopent-2-enol, cyclopent-2-enone and 2-methoxycyclopentanol for MeOH, 2-ethoxycyclopentanol for EtOH, 2-isopropoxycyclopentanol for *i*-PrOH and 2-(*tert*-butoxy)cyclopentanol for *t*-BuOH.

**Supplementary Table 5.** The results of CHN analysis.

| No. | Catalyst | C content ^a^ (wt.%) | C/N ^b^ |
| --- | --- | --- | --- |
| 1 | Ti-MWW | - | - |
| 2 | R-Ti-MWW-PI | 8.26 | 4.9 |
| 3 | R-Ti-MWW-PI-cal | - | - |
| 4 | Ti-MWW-AM | 11.12 | 5.2 |
| 5 | Ti-MWW-AT | 3.98 | 5.0 |

^a^ Determined by CHN analysis.

^b^ Calculated by C and N content (wt.%).
