# Supplementary figures and images for: Modified Ti-MWW Zeolite as a Highly Efficient Catalyst for the Cyclopentene Epoxidation Reaction
Source: Front Chem. 2020 Oct 9;8:585347. doi: 10.3389/fchem.2020.585347 (PMC7581914; doi:10.3389/fchem.2020.585347)

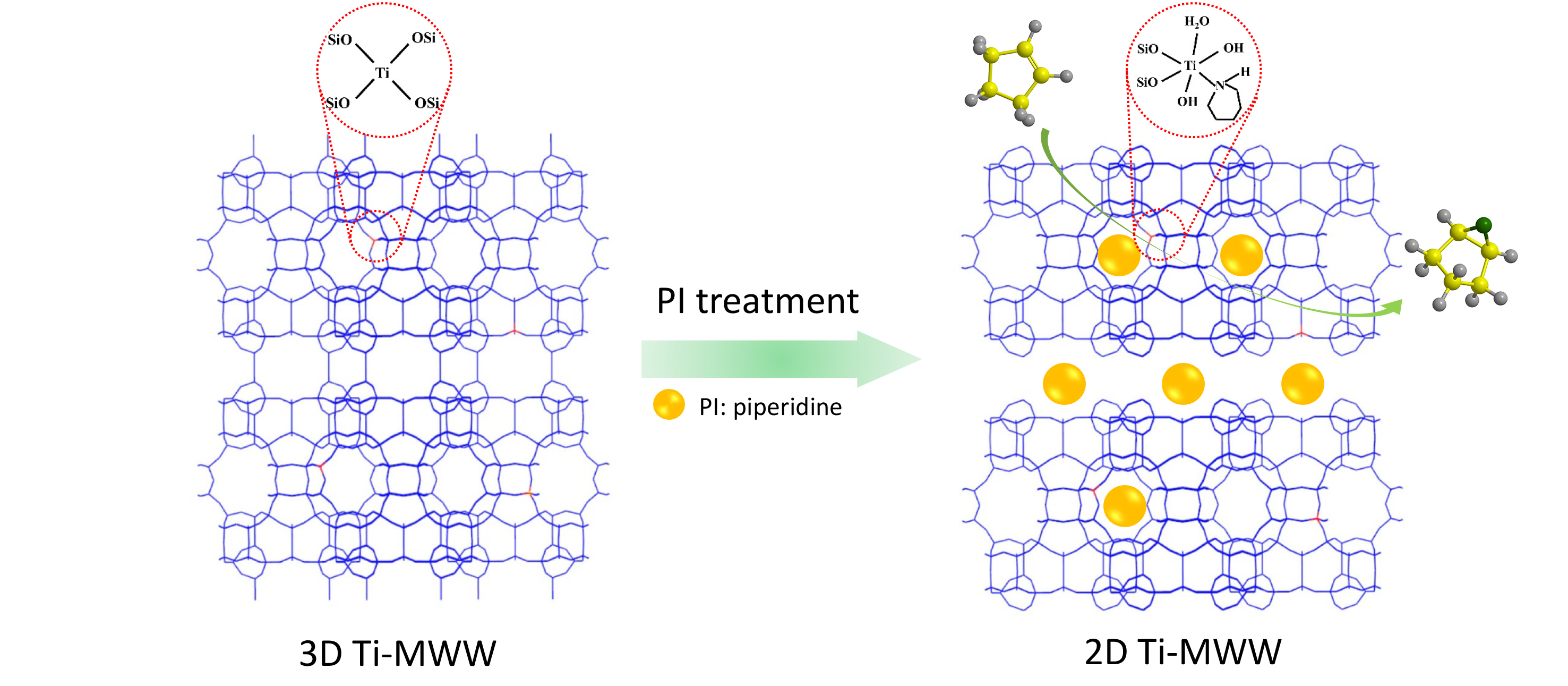

Supplement: Supplementary file 2 [file Image_1.JPEG]
